# Behavioral Observations in Northern UGANDA: Development of a Coding System to Assess Mother–Child Interactions in a Post-war Society

**DOI:** 10.3389/fpsyg.2019.02519

**Published:** 2019-11-07

**Authors:** Julia Möllerherm, Elizabeth Wieling, Regina Saile, Marion Sue Forgatch, Frank Neuner, Claudia Catani

**Affiliations:** ^1^Department of Psychology, Clinical Psychology and Psychotherapy, Bielefeld University, Bielefeld, Germany; ^2^Marriage and Family Therapy/Department of Human Development and Family Science, College of Family and Consumer Sciences, University of Georgia, Athens, GA, United States; ^3^Oregon Social Learning Center, Eugene, OR, United States

**Keywords:** behavioral observations, structured observation, post-conflict setting, mother-child dyads, cultural adaptation, parenting

## Abstract

There is growing interest in causes and consequences of disruptions in parent-child relationships in post-war environments. Recent studies mainly relied on self-reports to gain information about family dynamics following war exposure. Considering the limitations of self-report measures, we see the need for an in-depth examination of post-conflict parenting based on observational and quantitative data. The aim of the present study was the development of a coding system for a culturally bound description of parent–child interactions in northern Uganda, where virtually the entire population has been severely affected by 20 years of civil war. Interactions of 101 mothers and their 6- to 12-year-old children were observed during a structured interaction task (problem solving discussion). Foundation for the development of the coding system was the Family and Peer Process Code (FPP code). The cultural adaptation of the FPP code was based on in-depth qualitative analyses of the problem solving task, including a combination of inductive and deductive latent content analyses of textual data and videotapes, member checking and consultations of experts in the field of behavioral observations. The final coding system consists of 35 exhaustive and mutually exclusive content codes including codes for verbal, vocal, and compliance behavior as well as 14 affect codes. Findings indicate that the assessment of behavioral observations in post-conflict settings provides unique insights into culture- and context-specific interaction patterns and may be critical for the development and evaluation of parenting interventions.

## Introduction

Family dynamics and parent–child interactions have recently become the focus of attention in the research of child mental health in post-conflict settings (Catani et al., 2008b; Panter-Brick et al., 2014; Betancourt et al., 2015; Saile et al., 2015; Wieling et al., 2015). Nevertheless, little is known about specific associations between risk factors in post-war societies, parent–child interactions and children’s psychosocial adaptation and healthy development.

Our understanding of parenting and child development in post-conflict settings is guided by the Social Interaction Learning Model (SIL model; Patterson and Reid, 1984; Patterson, 2005). The SIL model posits that contexts (e.g., socioeconomic status, culture, social support, neighborhood) influence parenting and thereby indirectly influence child outcomes. Risk contexts (e.g., poverty, community violence) contribute to a decrease in positive parenting practices (positive involvement, skill encouragement, monitoring, effective discipline, problem solving skills) and an increase in coercive parenting practices. In distressed families, these coercive interactions escalate over time through negative reinforcement and lead to deviant behaviors in children (Forgatch et al., 2004), which in turn generalize across social settings of children (e.g., peers, school). Thus, the SIL model provides a theoretical framework to the understanding of the interaction between the individual person and different contexts. In doing so, it defines parenting practices as the most important and most proximal factor that shape children’s development and ongoing adjustment, even in highly aversive contexts.

Longitudinal studies in Western cultures provided empirical evidence for all components of the SIL model (Patterson, 2005; Patterson et al., 2010). However, to our knowledge there are no studies that examined the applicability of the SIL model to post-conflict settings. Despite the lack of empirical evidence, as outlined below, there seems to be some support for the relevance of the proposed mechanisms for child development in these environments.

Regarding the association between risk contexts and an increase in coercive parenting practices, previous studies in post-conflict settings suggest that war exerts a deleterious effect on family relationships and parent–child interactions (Haj-Yahia and Abdo-Kaloti, 2003; Catani et al., 2008a, 2009; Boxer et al., 2013). In Sri Lanka children‘s traumatic war exposure predicted child-reported victimization in the family (Catani et al., 2008a). Palestinian children who had been exposed to higher levels of political violence perceived elevated levels of strict discipline, rejection and hostility in parents (Punamäki et al., 1997). A recent study that examined war-affected families in Uganda found that traumatic war exposure of female caregivers as well as children’s own traumatic experiences were associated with child-reported experiences of maltreatment in the family (Saile et al., 2014).

In addition, there is evidence underscoring the importance of parent–child interactions for risk and resilience in children growing up in post-conflict settings. On the one hand, in post-conflict settings family dysfunction, domestic violence and poor caregiver mental health have been associated with increased psychopathology among children (Panter-Brick et al., 2011, 2014; Betancourt et al., 2013, 2015; Saile et al., 2015). On the other hand, authors emphasize that family relations and the quality of parenting are important resources in terms of resilience among children exposed to trauma and adversity (Masten et al., 1999; Gewirtz et al., 2008; Klasen et al., 2010; Masten and Narayan, 2012; Betancourt et al., 2014). Findings from cross-sectional studies in the Middle East showed that non-punitive, warm and supportive parenting styles mitigated the impact of war and military violence on social, behavioral and psychological difficulties in Palestinian, Sri Lankan, Israeli Arab and Jewish children (Qouta et al., 2008; Thabet et al., 2009; Dubow et al., 2012; Lavi and Slone, 2012; Sriskandarajah et al., 2015; Dekel and Solomon, 2016).

The above mentioned studies provide first indications of the proposed interplay between post-conflict settings, parenting and child development and thereby make an important contribution to the understanding of parenting in post-conflict settings. However, to our knowledge, studies to date have solely focused on parenting styles such as hostile, violent or supportive parenting. In addition, across studies the assessment of parenting quality solely relied on self-reports of children or parents on cross-culturally adapted questionnaires and event-checklists (Catani et al., 2008a, 2009; Dubow et al., 2012; Saile et al., 2015). Next to a number of methodological limitations of self-report measures (e.g., social desirability, retrospective reporting), most importantly, they do not allow for a moment-to-moment assessment of interactive behaviors. Thus, based on the current literature the following can only be insufficiently answered: What types of behaviors show that mothers are positively involved? What are methods parents in post-conflict settings use to encourage or discipline children? How do coercive parent–child interactions unfold in time and manifest? The way parenting dimensions are expressed varies across contexts and cultures (Bornstein, 2013). Therefore, a comprehensive understanding of these culture specific parenting practices and parent–child interactions is crucial to inform parenting interventions and thus, support children’s healthy development in post-conflict contexts.

Consequently, we see the need for an in-depth examination of parenting practices as well as interactional processes between parents and children in post-conflict environments bypassing the shortcomings of self-report measures. According to Gardner (2000) observational techniques are an advisable complementation of self-report measures since they facilitate the specification of behaviors of interest by the researcher rather than by the participating parent or child. Besides, they allow for a direct observation of interactional processes as they unfold in time. Thus, direct observations make context-specific, actual and genuine parenting practices and child behaviors accessible for research. In addition, behavioral observations are characterized by their potential to reliably identify children at risk of experiencing maltreatment (Kavanagh et al., 1988; DeGarmo et al., 2006), their capability for the development and evaluation of appropriate interventions (Aspland and Gardner, 2003) and their predictive validity (Patterson and Forgatch, 1995). Despite these benefits, to our knowledge behavioral observations of parent–child interactions have not yet been employed in post-conflict environments.

A prototypical example of such a post-conflict setting is northern Uganda where the present study took place. Parents there were affected by 20 years of civil war and their children still face the daily stresses of the transition phase. These include insufficient health care and education, inadequate access to water, land disputes, landmines and unexploded ordnance (United Nations Office for the Coordination of Humanitarian Affairs, n.d.), partner violence, alcohol abuse (Saile et al., 2013) and child neglect and maltreatment (Ministry of Gender Labour and Social Development, 2009; Saile et al., 2014). As parents are the primary agents of socialization, fostering positive and non-violent parenting in the transition phase may be an important component of the peace-building process. To achieve this aim a better understanding of parenting behaviors in this context is indispensable.

The current study was part of a larger project examining parenting in post-conflict northern Uganda. Even though fathers become increasingly important, traditionally child-rearing is still a women’s domain in Uganda (Nkwake, 2009). Thus, we decided to focus on mothers and their children. The objective of the current study was the description of the development of a context-sensitive coding scheme based on qualitative analyses and microcoding of behaviors shown by mothers and their children during a structured interaction task. We intended to add depth to existing work, enable greater understanding of context-specific parenting practices and establish a basis for further study of antecedents and consequences of parent–child interactions in northern Uganda. The structured interaction task chosen for this purpose was a problem solving discussion. We assumed that the observation of mothers and children resolving their own disputes would provide a virtually naturalistic account of maternal parenting strategies and child behaviors and therefore would facilitate an in-depth insight into interactional patterns associated with violent escalations.

## Methods

### Sampling

Participants were recruited through widespread verbal and flyer advertisements at schools, banks, churches, hospitals, markets, women’s groups, and Gulu University. Participants were purposely selected to include mothers from the Acholi ethnic group representing varying educational levels and degree of exposure to war-related stressors. In addition, only mothers were included who had a biological child aged between 6 and 12 years of age.

Out of the 132 women that initially showed interest in study participation, 106 mother-child dyads finally participated in the study. Reasons for non-participation included discomfort with being videotaped, different expectations regarding the study (financial support of the child, school fees) or being ineligible for participation (not biological mother, not Acholi, no child in the needed age range). For the present study, five mother-child dyads had to be excluded from the sample due to mothers not reporting any problem issue or children being too distressed to proceed following the discussion of the Negative Event so our analyses were based on a final sample of 101 mother-child dyads. Sample characteristics are displayed in Table 1.

**TABLE 1:** Sample characteristics of mothers and children.

**Mothers, *N* = 101**	***M* or %**	**(*SD*) or (*n*)**
Age, *M (SD)*	33.34	(6.63)
Marital status,% (*n*)		
Single/never married	9.9	(10)
Partner/cohabiting	35.6	(36)
Married	21.8	(22)
Divorced	17.8	(18)
Widowed	14.9	(15)
No. of children in household, *M (SD)*	5.53	(2.67)
Household possessions per capita, *M (SD)*	111.21€	(223.64€)
Highest educational level,% (*n*)		
No school	4.0	(4)
Some primary school	28.7	(29)
Completed primary school	4.0	(4)
Some secondary school	32.7	(33)
Completed vocational school	1.0	(1)
Completed O-level	17.8	(18)
Completed A-level	4.0	(4)
Some university	2.0	(2)
Completed university	5.9	(6)
Regular income,% (*n*)		
0 €	37.6	(38)
<30 €	19.8	(20)
<60 €	14.9	(15)
<90 €	12.9	(13)
<120 €	12.9	(13)
≥150 €	2.0	(2)
Occupation^a^,% (*n*)		
Small scale business/brewing alcohol	35.6	(36)
Teacher	16.8	(17)
Cleaning lady	12.9	(13)
Trader	9.9	(10)
Craftsman/tailor	5.9	(6)
Farmer	5.9	(6)
Government servant	3.0	(3)
Housemaid	4.0	(4)
Employee in a hotel	2.0	(2)
Employee in a non-governmental organization	2.0	(2)
Medical profession	1.0	(1)
Childcare	1.0	(1)
No occupation	4.0	(4)
Effects of war^a^,% (*n*)		
Displacement	88.1	(89)
Abduction	25.7	(26)
Close family member died during war	50.5	(51)
Mother’s previous victimization^b^, *M (SD)*		
Partner violence (CAS)	8.10	(5.93)
Trauma exposure (VWAES)	11.05	(4.47)
History of childhood family violence^c^	7.47	(4.21)

**Children, *N* = 101**	***M* or %**	**(*SD*) or (*n*)**

Age, *M (SD)*	8.92	(1.90)
Female,*% (n)*	47.5	(48)
Half-orphan,% (*n*)	16.8	(17)
Living together with both biological parents,% (*n*)	46.5	(47)
Children’s previous victimization^b^, *M (SD)*		
Trauma exposure (VWAES)	3.88	(2.85)
Family violence^c^	3.80	(2.67)

*CAS, Composite Abuse Scale (Hegarty, 2007). VWAES, Violence, War and Abduction Exposure Scale (Ertl et al., 2010). ^*a*^Data were collected based on non-mutually exclusive categories, thus values in row don’t add up to 100%. ^*b*^Mean number of different violent or traumatic events experienced. ^*c*^31-item checklist to measure adverse events experienced at home (Saile et al., 2014).*

### Materials

#### Observational Dyadic Interactions

Mothers and children participated in a structured interaction task, a problem solving discussion (Parent Issue). The task was part of a comprehensive behavioral observation including five structured interaction sections. For the present study, the problem solving discussion is the only relevant task. It had been pretested and culturally adapted in a pilot study in 2010 (Wieling et al., 2017). The remaining four tasks were comprised of two activity-oriented tasks focusing on cooperation between mothers and children and two emotion-focused discussions of one positive and one negative event from the child’s life. Each task took 5 min and was recorded on video. The order of the tasks was always the same; the problem solving discussion took place in the fourth place. Discussion topics were identified on the basis of mother’s responses to an adapted version of the Parent Issues Checklist (Rains and Corrigan, 2004) which comprises 27 issues that can lead to discord between parents and adolescents. Based on findings from the pilot study in the present study the Parent Issues Checklist was reduced to seven issues relevant to the Ugandan context and one open response item. The instruction for the Parent Issue was for mother and child to discuss the problem issue and to come up with a solution. The dyads most often discussed issues related to Keeping body clean (26.4%), Helping out around the house/chores (22.6%), Fighting with siblings/friends (12.3%), Getting up in the morning (8.5%), and School performance (6.6%).

#### Verbal Transcripts

In sum, 50 interaction tasks were transcribed verbatim and then translated into English by three local interpreters immediately following data collection. Out of these 50 transcripts the English versions of 20 randomly chosen transcripts were compared to the original videotaped conversation in the Acholi language. This accuracy check was done by an interpreter other than the one who originally translated and transcribed the interactions. Since satisfying accuracy was reached the remaining interaction tasks were directly transcribed into English, but still checked for accuracy by another interpreter. Regular meetings with the first author were held throughout the transcription process. Within these meetings the interpreters and the first author continuously discussed insecurities regarding translations of specific local idioms until agreement on standardized procedures could be reached. All transcripts included time counts corresponding to videos and mother-child verbal interactions.

### Procedure

Study procedures were approved by the ethics committee of the German Research Foundation (DFG), the ethics committee of Gulu University in Uganda and the National Council for Science and Technology (UNCST) in Uganda.

The study took place in the office of a humanitarian organization, which is called vivo Uganda and is located in Gulu town. Before the study started a team of 14 local trauma therapists and three local interpreters received a 2-day training in the collection of video-based behavioral observation data.

Women initially interested in study participation were invited together with their child to the study office. Upon their arrival mothers and children were provided with detailed information about the purpose and procedure of the study, potential risks, confidentiality and their right to withdraw at any time. Specific attention was paid to carefully explaining the procedure including the purpose of video-based behavioral observations to increase familiarity with the setting and to reduce anxiety. Mothers who continued to show interest in study participation were asked to provide written informed consent for themselves and their children’s participation in the study (signature or fingerprints). In addition, children’s assent was obtained.

Next, interviewer-assisted self-reports from mothers and children on sociodemographic data, parenting, family and partner violence, trauma exposure and psychopathology were obtained using standardized questionnaires. The analyses of these data and their relationship with observational outcomes are beyond the scope of the present paper and will be explored in future work. Mothers additionally responded to the Parent Issues Checklist. Mothers and children were interviewed separately by different local counselors in private rooms within the study office to create safe space, where both mother and child could talk freely.

After a short break behavioral observations were conducted in a different room within the study office. The room was equipped with items common in Uganda such as a traditional Ugandan mat for mothers and children to sit on. Apart from the camera and microphone mother-child dyads were left unattended during the time of each interaction activity.

The entire procedure including explanation of the study purpose, separate diagnostic interviews with mothers and children, and behavioral observations lasted for approximately 2.5–3 h. Participants did not receive any incentives for the study but were provided with snacks and reimbursement for travel expenses. At the end of the video observation a short debriefing was held. Mothers and children were asked how they felt and whether they had any further questions or concerns regarding their study participation. In cases where participants were distressed due to extremely harsh living circumstances (e.g., severe partner violence) or psychopathology (e.g., suicidality, post-traumatic stress symptoms) they were referred to our local trauma therapists, who offered counseling or trauma therapy.

### Cultural Adaptation of the Family and Peer Process Code

#### The Family and Peer Process Code

The Family and Peer Process Code (FPP code; Stubbs et al., 2001) is a synthesis of three closely related behavior codes that were developed over a period of 20 years by the Oregon Social Learning Center. It is a widely used coding strategy to capture behaviors of interest in family and peer interactions. We chose the FPP code as the foundation for our systematic cultural and contextual adaptation of codes because it is based on exhaustive and mutually exclusive microsocial codes that capture coercive and harsh as well as affectionate and caring interaction patterns as they unfold in time. The FPP code comes along with a training manual, which includes very detailed definitions of codes, decision rules and a huge variety of examples of associated behaviors. The FPP code permits simultaneous coding of different facets of behaviors, namely content and affect. Content describes an individual’s behavior as it changes over time. There are 24 content codes, eight of which are defined as positive, nine as negative, and seven as neutral. They are further divided into verbal, vocal, non-verbal, physical, and compliance behaviors. Verbal behaviors are again divided into the dimensions Conversation, Interpersonal, Strong interpersonal, Directives, and Responses to directives. Affect is defined by six affect codes: Happy, Caring, Neutral, Distressed, Aversive, and Sad. These emotional states are rated based on facial expressions, tone of voice, and body language.

#### Adaptation of the Coding Manual

The adaptation of the FPP code for the Parent Issue followed four stages. The first two stages were dedicated to a combination of inductive and deductive latent qualitative content analyses (Graneheim and Lundman, 2004; Elo and Kyngäs, 2008) of the textual and observational data to identify culture-specific parenting practices and child behaviors. Throughout the analyses an audit trail of documents and memos was kept to reflect decisions and changes along the way. First, we began with an inductive latent qualitative content analysis of the transcripts. This analysis was done in close collaboration between Dr. Wieling, two doctoral students of the University of Minnesota and the first author of this paper over a period of 6 weeks. We started by coding a randomly chosen subsample of 10 transcribed Parent Issues. After getting immersed in the data by reading through the transcripts several times, we coded emerging units of meaning following a line-by-line open coding approach. After coding all transcripts separately, we met to review our findings, highlight similarities, discuss differences in our interpretations of mother and child verbal behaviors, and formulate inductive categories. This process was repeated until no new units of meaning emerged and we agreed that mother and child behaviors were well described by the developed categories (thematic saturation; O’Reilly and Parker, 2013). In the second stage initial categories were compared to definitions of the original content codes rating verbal, vocal, and compliance behaviors as well as the coding structure of the FPP coding manual. Initial categories were further specified and formulated into codes, new codes were developed and already existing codes were culturally adapted. This process was mainly accomplished by the first author and Dr. Wieling. However, we continued to conduct regular meetings within the whole team to compare, discuss, and reconcile differences. Following a procedure similar to the aforementioned qualitative content analysis we analyzed a series of randomly selected videotapes to develop and adapt affect codes as well as content codes that captured non-verbal and physical behaviors. This process of analysis included consultations with three experts in the field of behavioral observations and affect coding.

Member checking is a validation technique in qualitative research that combines a number of different approaches to explore the credibility of results (Birt et al., 2016). The primary objective of this method is to gain feedback of participants on results in order to check accuracy and resonance of findings. In the third stage we applied an approach similar to member check focus groups (Klinger, 2005; Birt et al., 2016). About 4 months after the completion of data analyses the first author went back to northern Uganda for a period of 7 weeks to review and verify findings and decisions concerning the adaptation of the coding manual. In contrast to common approaches of member checking, we did not directly consult with participants of the study. Due to ethical (e.g., confidentiality) and logistical constraints and limited resources, only members of the local staff of vivo Uganda participated in the discussion of the applicability of the developed coding manual. The local team included four female trauma therapists and two male interpreters. Three of the four female trauma therapists were mothers themselves. The male interpreters were young men who still stayed together with their guardians, had no children of their own, and recently started their studies at Gulu University. All team members were Acholi. Their working experience as counselors or interpreters with vivo Uganda ranged from about one to almost 10 years.

Before the implementation of the actual workgroups, we discussed personal views of our local team members on family values, roles and responsibilities of family members, and potentially expected biases in analyzing and interpreting mother and child behaviors. This approach was used to consider inter-individual differences between members of the Acholi ethnic group reflective of their personal familial backgrounds and professional experiences as counselors and interpreters.

Workgroup discussions were based on videotapes as well as statements from transcripts. All workgroup discussions were audiotaped and transcribed for documentation purposes. Workgroup discussions based on videotapes were led by a number of open, non-directive questions about the previously watched mother–child interactions. These questions referred to the identification of emotional expressions, the interpretation and cultural meaning of non-verbal and physical interactive behaviors as well as impressions and views on the quality of the problem solving process and the overall mother–child interaction quality. In addition, workgroup discussions were employed in order to verify our interpretation of mother and child verbal behaviors. Local team members were handed statements from transcripts and asked to write down their view on the cultural meaning of these statements and their assumptions regarding the intention of the speaker as well as probable verbal, emotional and non-verbal responses to the statement by the interacting partner. Notes were then discussed within the group. Findings from workgroups were used to further specify the adapted coding scheme.

Finally, Dr. Wieling and the first author developed guidelines for training and data entry procedures. Based on these guidelines two teams of in total seven graduate students from the Bielefeld University and the University of Minnesota were trained in the application of the newly developed coding system during weekly meetings over a period of 2 months each. These meetings were used to test confirmability of the developed coding system and inter-coder agreement within a group of Western observers. Trainings were based on the very same textual and observational data from the Parent Issue that were used during the previous adaptation of the coding manual. Findings were employed to modify code definitions and decision rules where necessary.

## Results

### Main Findings Guiding the Adaptation Process

In the following section, only some of the main findings guiding the adaptation process will be presented, as a more detailed description would go beyond the scope of this paper.

#### Maternal Verbal Behaviors

Content analysis and workgroup discussions revealed that there was little mutual verbal exchange between the majority of mothers and children. In fact, in most dyadic interactions mothers highly lead the discussion by advising their child, stating behavioral expectations, teaching morals and values, revising wanted behaviors, and asking rhetorical questions. However, a few mothers invited their child to share his or her view on the problem issue and it‘s resolution by asking open questions and validating children’s responses.

To account for these interaction patterns we developed the dimension Teaching strategies, which consists of codes for advising behaviors as well as codes to capture maternal attempts to actively involve children in the problem solving process. Especially with regard to the latter, workgroup discussions revealed that some developed codes subsumed under Involving child in the Discussion required further modification in the coding manual. For instance, the code Exploration is defined by open questions to find out about children‘s thoughts, feelings, views, living circumstances, conditions, and reasons for misbehavior. Local team members agreed that examples for the first types of open questions actually expressed interest in the child and invited child participation. However, examples for open questions to find out about reasons for misbehavior were often, but not always, interpreted as coercive questions. One female trauma therapist explained this by stating: “this is a dangerous question, children are supposed to behave in a specific way and if they don’t do it then there is no excuse as to why they don’t do it; so whether the child is saying the truth or not, there will be punishment.” So most children would rather decide to keep quiet or respond by saying “nothing,” either out of fear or since they wouldn‘t feel invited to share their opinion. Further discussions revealed that not the question itself was considered to be coercive, but rather the way it was expressed. Thus, our local team members suggested other indicators to more easily differentiate between coercive questions and Exploration, which were added to the coding manual. These indicators included, amongst others: mother adds statements of concern, explains that there is no need for the child to fear any consequences and doesn‘t use any blaming words.

Behaviors subsumed under Advising Child were allocated to the two subdimensions Explanations and Expectations. Explanation codes capture statements that facilitate successful execution of the requested task and insight into the necessity of the task performance. Expectation codes comprise general evaluations of child behaviors as good and bad, but lack enough detail to facilitate successful future compliance by children. Our local team members also noticed these difference in the quality of advice giving, but didn‘t attach much importance to it. In fact, they stressed that all statements subsumed under Advising Child were indicators of concerned, responsible and caring mothers. Nevertheless, we decided to keep these different codes in order to allow for future thorough analysis of advice giving.

Regarding coercive and warm parenting practices we observed an uneven distribution. While harsh and blaming statements were used frequently, mothers infrequently expressed warmth or affection toward their children. In order to adequately capture positive and coercive verbal behaviors displayed by mothers we basically adopted codes from the original FPP coding manual that defined warmth or negativity of mother’s verbal communication. In addition, we extended the original FPP coding manual by a few codes to comprehensively define and capture coercive behaviors of the Ugandan mothers.

Our local team members largely agreed with the previously developed code definitions for coercive behaviors and consistently rejected them as negative parenting practices. For instance, the different intensity of coercive statements coded Negative interpersonal (negative evaluations of a person(s) behavior, appearance, state or conditions related to a person present) and Verbal attack (negative personalized and unqualified evaluations of a person present in the session) was similarly seen and statements were reliably identified by our local team. Whereas examples for Negative interpersonal were rated as blaming and criticizing statements, examples for Verbal attack were interpreted as insults or verbal abuses that comprised a negative evaluation of the person addressed (female trauma therapist: “This is a direct kind of blame, mother is saying that the child is always like this.”) However, the workgroup discussion of the code Threats changed our previous understanding of its meaning. Threats is a code that captures threatening, fear-installing consequences to future misbehavior. Even though our local team members agreed that most statements subsumed under this code may lead to fear in children, some also mentioned that it showed mothers’ attempt to protect their children (female trauma therapist: “It is a strong warning the mother is giving the child. It also shows that the mother is concerned about the future of the child.”) Based on further workgroup discussions on the implication and differentiation of stated consequences, the code Threats was refined and allocated to Teaching strategies.

#### Child Verbal Behaviors

In most dyads mothers were the initiator of the verbal interaction. The majority of children showed very little verbal participation in the communication with their mothers. Most of children’s talking comprised socially desired responses to mothers’ questions and teaching as well as unqualified affirmation to statements by mothers. Based on content analysis and workgroup discussions with our local Acholi team members we were able to differentiate between different types of child reactions: A subgroup of children anxiously refused most of the requested responses. Most children displayed respectful listening and socially desired responses, which our local team members described as normative child behavior in the Acholi culture. However, there was also a group of children who felt free enough to openly share experiences and even ask self-initiated questions, e.g., regarding the purpose or surrounding of the interaction activity. Based on workgroup discussions with our local Acholi team members we drew the conclusion that self-initiated and free verbal participation by children in the problem solving process would picture a rather free and positive relationship to their mothers. However, we concluded that slightly oppositional statements would be perceived as disrespectful child behaviors.

Concluding, the original FPP code manual needed to be adapted to facilitate the distinction between socially desired and self-initiated statements by children as well as a differentiation of the connotation of self-initiated statements by children. To achieve this in the adapted version of the FPP code we applied child content codes that reflected statements with positive, neutral or negative connotation only when expressed statements were clearly self-initiated. Additional content codes were included in the manual to capture positive, neutral or negative, but socially desired and expected responses to revising utterances, exploration or brainstorming, rhetorical, blaming or coercive questions (Neutral talk-responded, Affirm) as well as to capture freely expressed responses by children with a negative, aversive connotation (Bargain, Disagree/Talk back).

#### Coding of Mother and Child Affect-Related Behaviors

In the Ugandan sample mothers and children displayed little overt positive or negative facial and emotional expressions as defined by the original FPP code manual. A significant number of mothers seemed to highly control their voices resulting in an even-tempered, moderate tone of voice. In addition, facial expressions were not clearly visible in all cases due to the use of only one camera and the lighting conditions. On the basis of qualitative analyses and workgroup discussions with our local team members we identified a series of culturally specific indicators of affect-related behaviors. In this regard, behaviors of interest were eye-to-eye contact, sitting position, posture, body movements, and physical interactive behaviors. For instance, we noticed that a significant number of children avoided eye-contact with their mothers (e.g., looking down at the floor for almost the entire 5 min interaction) except for occasional gazes. Our local team members emphasized that eye-to-eye contact between elders and children is relatively uncommon in northern Uganda and that the avoidance of mutual eye contact with mothers can be interpreted as an indicator of respectful, attentive and positively involved child behaviors. However, they presumed that a reliable identification of the quality of children’s involvement would need to be based on additional indicators as well. For instance, the avoidance of mother’s eye-contact in combination with a frozen posture or nervous body movements, spatial distance toward the mother while sitting, and frightened winces when touched by mother was presumed as indicative of children’s anxiety or distress. In contrast, children’s avoidance of mutual eye-contact combined with behaviors such as actively moving away from the mother, turning the back toward mother, and verbally responding in a defiant manner was considered aversive, disrespectful child behaviors.

In sum, our findings suggested that culturally decisive nuances of emotional expressions and affect-related behaviors were defined by the composition of a number of non-verbal and physical interactive behaviors rather than facial expression and tone of voice alone. Thus, we decided to aggregate indicators of positive and negative emotional expressions as well as non-verbal and physical interactive behaviors to comprehensively and globally define the quality of affect-related behaviors displayed by mothers and children. This approach is in contrast to the original FPP code manual that differentiates between a series of codes to separately rate affect as well as non-verbal and physical behaviors.

### Adapted Coding System

#### Final Content Codes

The resulting coding scheme consists of 35 exhaustive and mutually exclusive content codes to rate verbal, vocal, and compliance behaviors. Twenty of the content codes were restructured or newly added for the Ugandan sample. Definitions of the remaining content codes were slightly adapted and examples deriving from the Ugandan data were added to the manual. Out of the 35 content codes, seven are defined as positive, 17 as neutral, and 11 as negative. Content codes are further differentiated by their applicability for mother and/or child behaviors (e.g., teaching behaviors are only applicable for mother behaviors).

With respect to the temporal resolution of codings our level of analysis is an event-based coding system: new codes are entered each time the verbal or vocal content of the participant changes. Therefore, the behaviors that were displayed can be interpreted sequentially, on the level of frequencies of specified behaviors as well as behavioral contingencies (e.g., compliance contingent on command).

Table 2 provides a comprehensive overview of the resulting content codes, statistical indices of those content codes (mean value of the number of counts of content code across the 5-min interaction and percentage of participants who qualified for content code at least once), and examples of associated verbal behaviors deriving from the Ugandan data. As can be seen from Table 2, next to neutral verbal remarks (*M* = 6.85) mothers frequently made negative interpersonal statements (*M* = 3.97), asked coercive questions (*M* = 3.22), and demanded affirmation from their child (*M* = 6.21). Positive interpersonal remarks were rather rare (*M* = 1.23). Also with regard to the frequency of the applied teaching strategies we found an uneven distribution. Mothers prevalently stated behavioral expectations (*M* = 6.25), whereas detailed explanations of requested behaviors (e.g., Explaining WHY to do task: *M* = 3.09) or open questions to involve children in the conversation (e.g., Brainstorming: *M* = 0.45) were less frequently used. Children mainly made neutral remarks (*M* = 11.03), mostly in direct response to their mothers (*M* = 8.16).

**TABLE 2:** Overview of the adapted content codes, basic descriptions of associated behaviors, and statistical indices of the adapted content codes.

**Content codes**	**Basic descriptions**	**Statistical indices**					
		**Mother**			**Child**		
		***M ^a^***	**(*SD*)**	**%^b^**	***M ^a^***	**(*SD*)**	**%^b^**
**Conversation**							
Positive talk	Positive verbal expressions directly related to person(s) outside the session or objects, possessions, situations, occurrences, living circumstances, and preferences: “These days you see life is easy.”/“That biscuit is sweet.”/“Okwi’s shirt is good, it fits him.”	0.08	(0.30)	6.9	0.11	(0.45)	7.9
Neutral talk-self-initiated^c^	General conversational verbal interaction: “Mother, all the floor of the house is cemented.”/“They are coming back.”/“Is this where they get water?”	6.85	(3.91)	100.0	2.87	(4.56)	55.4
Neutral talk-responded^d^	Socially desired and expected verbal responses or statements by children: (Mother: “So is it good if you come back home late?”) Child: “It is not good.”	–	–	–	8.16	(7.85)	90.1
Negative talk	Negative counterpart of Positive talk: “There were those days when your father was chasing us, and we had to sleep in the bush.”/“There are some boys who like raping young girls.”/“This video is taking a very long time!”/“My waist hurts.”	0.50	(0.82)	33.7	0.82	(1.88)	26.7
Demanding affirmation^d^	Rhetorical questions where mothers repeatedly request their child to affirm what they said (vocal and verbal statements are combined here).	6.21	(6.35)	91.1	–	–	–
**Interpersonal**							
Positive interpersonal	Positive evaluations of a person(s) behavior, appearance, state or conditions related to a person present in the session including thanking: “Thank you for telling me that you will wake up early!”/”Forgive me for quarreling at you.”/“I will wash your clothes if you are sick.”	1.23	(1.98)	52.5	0.09	(0.38)	6.9
Positive interpersonal consequences^d^	Positive, motivational relational consequences: “I’ll do something good for you if you read your books.”/“If you wake up early it makes me very happy!”	0.19	(0.58)	11.9	–	–	–
Tease	Verbal jokes or humor addressed to self or someone in the observation including banter, playful pestering, and gentle wit directed at others.	0.10	(0.46)	5.9	0.04	(0.23)	3.0
Negative interpersonal	Negative counterpart of Positive interpersonal “Why are you so stubborn like that, when I tell you to do something you refuse?”/“I always tell you not to beat Opiyo, but when I come back home I immediately hear that you have been fighting again.”/“Here is not the place for you to look angry!”	3.97	(3.81)	82.2	0.14	(0.44)	10.9
Negative interpersonal consequences^d^	Negative, guild-installing relational consequences: “You need to come back early so that you don’t hurt my feelings.”/“I just search for money and you don’t want to get things, this makes me so sad.”	0.80	(1.29)	42.6	–	–	–
Coercive questions^d^	Rhetorically (often repeatedly) asked aversive questions that don’t allow for any genuine response by children, but mainly intend to make the child agree that he/she showed inappropriate behaviors: “Do I always beat you without you doing any wrong?”/“Don‘t I give you time to play?”	3.22	(4.56)	65.3	–	–	–
**Strong interpersonal**							
Endearment	Positive personalized and unqualified evaluations of a person present in the session: “I love you.”/“You are beautiful.”	0.23	(0.60)	15.8	0.14	(0.53)	8.9
Self-disclosure	Statements that reveal important information about the speaker including family experiences that directly affect the child/person. These can be descriptions that are not always directly observable in the course of day-to-day interactions with others.	0.35	(0.70)	24.8	0.31	(0.83)	18.8
Verbal attack	Negative counterpart of Endearment: “You are just a dirty child!”/“You are a liar!”	0.23	(0.84)	10.9	0.01	(0.09)	1.0
**Vocal**	Laughing and neutral vocal utterances.	2.81	(3.20)	80.2	1.60	(2.38)	53.5
**Teaching strategies^d^**							
**Involving child in discussion**							
Exploration	Open questions to find out about children’s thoughts, feelings, views, living circumstances, conditions, and reasons for behaviors: “What makes it difficult for you to wake up early in the morning?” [Differentiation: Negative interpersonal (“Why are you so stubborn?”)]	1.85	(2.44)	60.4	–	–	–
Brainstorming	Open questions that invite the child to share suggestions on how to solve the problem: “What can we do to make fetching water easier for you?” [Differentiation: Threats (“What should I do to you if you don’t do it?”)]	0.45	(1.21)	18.8	–	–	–
Subsequent questions	Non-rhetorical, closed questions or vocal utterances that follow open questions and clearly show further interest in what children want to share (vocal and verbal statements are combined here).	4.72	(5.01)	84.2	–	–	–
**Parental validation**	Repetition/Rephrasing of statements in order to confirm that mothers understood what their child was saying, to assure that they are listening and/or to clarify that they understood their child correctly: (Child: “Me alone.”) Mother: “You look at the sign post alone?” (Differentiation: Coercive questions)	2.41	(2.93)	71.3	–	–	–
**Threats**	Threatening, fear-installing consequences to future misbehavior that imply physical, emotional or psychological harm: “Now, if I find you fighting each other then I start beating all of you.”/“You will die if you don’t keep your body clean.”/“If you continue to behave like that, then there might come a time when I leave you.”	0.73	(1.49)	33.7	–	–	–
**Advising child**							
**Explanations**							
Explaining HOW to do task	Detailed examples or explanations of how child will be able to successfully accomplish requested task or behave in an appropriate way: “Early in the morning, you wake up, wash your face, you brush your teeth, you wash your legs thoroughly, you put on your uniform, you get your books, you get food and eat, and you go to school.” (Differentiation: Behavioral expectations)	3.87	(3.28)	86.1	–	–	–
Explaining WHY to do task	Explanations that teach children the importance of carrying out a specific task or show a specific behavior: “If you take long sleeping that means you will reach at school late and you will be given punishments like slashing the compound and the teacher would be teaching while you slash. That means you will miss the lessons and if you copy what has been taught from your friends you won’t understand because you missed the teacher’s explanations.” (Differentiation: Threats, Interpersonal consequences)	3.09	(3.31)	77.2	–	–	–
Revising explanations	Open questions or suggestions by mothers that revise, refresh or repeat what children should already know (regarding HOW and WHY to do task) by inviting child’s verbal participation.	2.75	(4.46)	53.5	–	–	–
**Expectations**							
Behavioral expectations	Stating expectations for child‘s behavior including what mother wants or thinks the child should or shouldn‘t do (statements don‘t facilitate successful compliance by the child): “You should be a respectful child.”/“Don‘t be a child who disagrees when talked to.”/“I want to find that all the time your body is clean.”/“You as a child are given birth to, to be sent.” (Differentiation: Explaining HOW to do task, Negative interpersonal)	6.25	(4.17)	94.1	–	–	–
Behavioral evaluations	General behavioral consequences and behavioral evaluations as good or bad that lack enough detail to facilitate comprehensive insight into the necessity of the task performance: “If your body is dirty, your brain will not be smart.”/“If you wake up early you will be a sensible child.”/“Fighting is bad.” (Differentiation: Explaining WHY to do task)	1.83	(1.92)	70.3	–	–	–
Revising expectations	Rhetorical/revising questions regarding good and bad behaviors as well as general, non-explanatory consequences to positive or negative behaviors: “Is stealing good?”/“If you do everything I tell you, what kind of child will you be?”/“If you are punched and killed, is it good?”	0.66	(1.42)	30.7	–	–	–
**Probing for compliance**	Questions where mothers ask their child if he/she will show the requested behavior in future: “So starting from today will you wake up early?”/“Next time if I teach you, will you repeat what I taught you?” (Differentiation: Coercive questions)	1.26	(2.00)	48.5	–	–	–
**Responses**							
Agree^c^	[Child: Can you tell me about grandma?”] Mother: “I will tell you about her, but first we talk about cleanliness.”/(Mother: “Starting today, will you wash plates?) Child: “Yes, I will wash them very, very clean and will even place them outside to dry.”	0.43	(0.93)	25.7	0.42	(0.93)	24.8
Affirm^c,d^	Behaviors that indicate attentive and affirmative listening through the use of vocal or verbal cues (e.g., “Ayaa”/“Ehng”/“Ooh”/“Mmm”).	8.07	(7.51)	95.0	13.40	(12.13)	95.0
Bargain^d^	A response where the child is challenging the mother or trying to reach a certain goal (e.g., getting incentives).	–	–	–	0.46	(1.87)	9.9
Disagree/Talk back^c,d^	Coded if a person present doesn’t agree to statements or suggestions of an interaction partner. Further it applies if children respond to statements by mother in a bold, defiant or disrespectful way. (Differentiation: Negative interpersonal)	0.38	(0.72)	28.7	0.64	(1.64)	23.8
Refuse^e^	Includes both explicit verbal response and implied verbal response to a directive indicating that one will not comply or grant permission.	–	–	–	–	–	–
**Command**	Firm directives, questions or requests for behavior changes potentially observable within the context of the observation: “You speak faster, time is running.”/“They want that we talk in a way that our voices are heard.”/“Sit properly!”	3.27	(3.67)	76.2	0.12	(0.43)	8.9
**Compliance behavior**							
Comply	The act of clearly obeying another’s request or command. Compliance is double coded with actual compliant response.	–	–	–	2.20	(2.57)	66.3
Non-comply	Any act of clearly disobeying another’s request or command. Non-compliance is double coded with actual non-compliant response.	–	–	–	0.95	(2.10)	35.6

*^*a*^Mean value of the number of counts of content code across the 5 min interaction. ^*b*^Number of participants who qualified for content code at least once. ^*c*^Applicable for mother and child behavior, but with differing connotation. ^*d*^Re-structured or newly developed codes. ^*e*^Adopted from original manual even though code never matched displayed behaviors. Missing numerical values indicate that the code is not valid for the corresponding participants.*

#### Final Affect Codes

Affect is coded following a macro coding approach in which affect displayed by mothers and children is coded once for each 5 min interaction task. This approach was used because mothers and children showed hardly any affective change across the 5 min interaction. Affect displayed by participants was categorized on the basis of 6 affect codes for mothers and 8 affect codes for children. For mothers overall affect was coded as Caring or Happy in one-fourth of cases, as Positive-controlled/nervous in about one-third, and as Aversive or Emotionally-detached in more than one-third of cases. The overall affect of half of the children was coded as internalizing affect-related behaviors, one third as Attentive or Happy, and about one-sixth as coercive child behaviors.

Tables 3, 4 provide an overview of the resulting affect codes, absolute and relative frequencies, and descriptions of associated affect-related behaviors.

**TABLE 3:** Overview of maternal affect codes, statistical indices, and basic descriptions of associated behaviors.

	***n*^a^**	**%*^b^***	**Basic descriptions**
1. Caring	24	23.8	Code is assigned to mothers who display warmth in the interaction with their children. Coding is based on body language and sitting position (e.g., body is facing the child, mother sits close to the child, lowers her upper body and leans toward the child), facial expression (e.g., warm, smiling, mother looks at the child and aims to keep eye contact), calm or gentle tone of voice, physical positive, non-intrusive interactive behaviors (e.g., carefully touching the child, checking cleanliness of child‘s body, offering a handkerchief when child’s nose is running), responsiveness toward child, and patience (e.g., constantly staying in contact with child while patiently waiting for child’s response).
2. Happy	1	1.0	As opposed to Caring, this code is assigned to mothers who additionally display playful behaviors and more openly express positive affect (e.g., laughter and giggling, clapping hands in excitement).
3. Positive-controlled/nervous	32	31.7	Mothers qualifying for this code display little self-initiated involvement in the interaction with their children (e.g., short duration of verbal interaction), highly control emotional expressions (e.g., nervous laughing, whispering or low voice) and show insecurity regarding the observational setting (e.g., nervous body movements, repeated glances at the camera). However, they display responsiveness and no indication of negative attitudes toward their children.
4. Neutral	8	7.9	Maternal affect-related behavior is of even-tempered quality and therefore doesn’t qualify for any of the other affect codes.
5. Emotionally- detached	17	16.8	Mothers assigned to this code show very little involvement, no or negative responsiveness, sit apart from their child, pay very little attention to their child, show irritation and boredom regarding their child.
6. Aversive	19	18.8	Code is assigned to mothers who display high involvement as well as an aggressive attitude and superior position in the relationship with their child. Coding is based on sitting position, duration of talking, facial expression (e.g., raised eyebrows, tense face, disapproving), tone of voice (e.g., harsh, loud, screaming), physical interactive behaviors (e.g., menacing gestures, raised forefinger while teaching, pointing at the child, hitting or slapping the child, throwing a handkerchief toward the child while child is crying), body language (e.g., looking at child from above to indicate superiority), and inpatient behaviors (e.g., interrupting child while talking, constantly changing commands referring to how child should sit, talk, wear his/her dress).

*^*a*^Absolute frequencies. ^*b*^Relative frequencies.*

**TABLE 4:** Overview of Children’s Affect Codes, Statistical Indices, and Basic Descriptions of Associated Behaviors.

	***n*^a^**	**%*^b^***	**Basic descriptions**
1. Attentive	23	22.8	Code defines affect-related behaviors similar to maternal behaviors rated Caring.
2. Happy	8	7.9	Code defines affect-related behaviors similar to maternal behaviors rated Happy.
3. Nervous	30	29.7	
4. Sad	8	7.9	
5. Distressed	14	13.9	
			Codes 3.-5. refer to internalizing affect-related child behaviors. They are defined on the basis of varying characteristics of behaviors such as: avoidance of maternal eye-contact (e.g., lowered head and looking down at the floor, looking around in the room, stares in directions other than where the mother is seated), very limited or anxious responsiveness toward mothers (e.g., anxious looks at the mother if requested to respond, long duration until verbal response, refusal of verbal response, frightened wince when touched by mothers), tone of voice (e.g., low tone of voice, stuttering, shaky voice), anxious or sad facial expressions (e.g., wide open eyes, blank stares, fighting tears, crying), body language (e.g., nervous body movements, shaky or cramping hands, frozen in their sitting position), and sitting position (e.g., attempts to sit away from mother, sitting position as expected by mothers).
6. Distracted-active	7	6.9	
7. Self-defensive	9	8.9	
8. Aversive	2	2.0	
			Codes 6.-8. represent coercive child behaviors. They are rated on the basis of varying characteristics of the following behaviors: body movements (e.g., lying on the floor, actively moving away from mother or turning the back toward her, running around in the room, acting out in front of the camera), tone of voice and emotional expression (e.g., normal or heightened tone of voice, sarcastic, grumpy, defiant, bored), decelerated or aversive responsiveness toward mother (e.g., laughing as a response to maternal requests, ignoring maternal requests), little mutual eye-contact.

*^*a*^Absolute frequencies. ^*b*^Relative frequencies.*

## Discussion

The aim of the present study was the development of a coding scheme for a culture-bound description of behaviors and emotions displayed by northern Ugandan mothers and their children during a problem solving task.

The main finding of the development of the coding manual was that the established content and affect codes to rate maternal and child behaviors represented aspects of behavioral dimensions found across cultures. For instance, we identified content codes that reflect core parenting dimensions also labeled warmth (Positive interpersonal behavior), rejection (Negative interpersonal behavior), autonomy support (Involving child in the discussion), and structure (Advising child) (Skinner et al., 2005). Actually displayed behaviors allocated to the respective content and affect codes, however, were in part contextually and culturally specific. Thus, in line with current literature (Bradley and Corwyn, 2005; Bornstein, 2013) we determined evidence for the necessity of comprehensive culturally anchored investigations of actually displayed behaviors, their meaning and function to prevent false conclusions due to the direct application of Western concepts to post-conflict societies.

First, we found that observed behaviors were different, but still connoted the same meaning and function as in Western societies. For instance, in Western cultures warmth is commonly defined by the expression of love, affection, caring, enjoyment, appreciation, emotional availability and support (Skinner et al., 2005). Mothers in the present sample, however, did not show open affection or emotional support. Instead, they expressed concern about their children’s material, scholastic and health-related needs and showed appreciation when children behaved well. Interestingly, a similar pattern was found in a study in which youths of 12 different cultures were asked which parental behaviors they perceived as supportive (McNeely and Barber, 2010). In comparison to the other ethnic groups, black youths from Cape Town more highly valued instrumental support, whereas they had the lowest rates regarding emotional and companionate support and allowance of freedoms.

Second, we found the same behavior patterns as in Western cultures that, however, differed in their meaning and function. One example is the meaning of mutual eye contact. In contrast to Western cultures, where the avoidance of direct eye contact is usually interpreted as a sign of discomfort, our local team members stated that it was an indication of respect when children looked down at the floor while talking to their mothers. Cultural differences in the meaning and indicative value of eye-to-eye contact were also found in a national, multi-site study conducted in the United States which aimed at developing cross-culturally valid guiding principles for the assessment of parent–child interactions (Bernstein et al., 2005).

Finally, we found substantial differences in the frequency of occurrence of content codes which seemed to be culturally and contextually specific. In a comparative study across eleven cultures, Barber et al. (2005) found comparable mean values for parental behavioral control and parental support. The mean values for parental psychological control were also comparable across all ethnic groups, but significantly lower than the other two parenting dimensions. In contrast, in the present study we observed that mothers exhibited controlling behaviors very frequently as opposed to supporting and warm behaviors. For instance, this was reflected in the mean values of codes that were allocated to Advising child (e.g., Explaining HOW to do task: *M* = 3.87) compared to Involving child in the discussion (e.g., Exploration: *M* = 1.85) as well as coercive (e.g., Negative interpersonal: *M* = 3.97) compared to warm parenting behaviors (e.g., Positive interpersonal: *M* = 1.23).

One possible explanation for the differences found could be that positive parenting is shown differently in northern Uganda as compared to other countries. Accordingly, advice giving in this context could be a sign of supportive rather than controlling parenting behavior. This is supported by the findings from the workgroup discussions. Whereas the international team, in particular the first author, unintentionally highlighted parenting practices that facilitated active involvement of children (most likely due to their Western cultural backgrounds), our local team members valued these parenting practices far less. In fact, they emphasized the importance of advice giving as one important aspect of positive involvement of mothers. This assumption is also supported by a qualitative study conducted with caregivers and children in three districts in Uganda (Central, Western, Northern) to identify community perceptions of protective and harmful parenting practices (Boothby et al., 2017). Authors found that across districts the aspects advises children and disciplines with violence were viewed as positive child-rearing. So there is reason to believe that the high amount of advice giving found in the present study is rather an indication of cultural influences, than a reflection of disruptions in parenting practices due to the post-conflict environment. However, the detailed analyses and description of different types of advice giving can facilitate further in-depth examination of potential associations between war-related adversities, quality of advice and child outcomes.

The high frequency of coercive as compared to warm parenting practices is in line with studies showing an increase in harsh parenting practices in post-conflict settings (Haj-Yahia and Abdo-Kaloti, 2003; Catani et al., 2008a, 2009; Boxer et al., 2013). Thus, in accordance with the theoretical framework of the SIL model, this finding might point at disruptions in parenting practices due to adversities in the post-war environment. However, to substantiate this assumption, future studies should investigate associations between the developed coercive and warm content codes, war-related stressors and children‘s adaptation.

Regarding an overall evaluation the greatest strength of the current study was the collaboration with an international workgroup and, above all, the involvement of local team members throughout the study and the development of the coding scheme. Regular reconciliation meetings within the international team were not only crucial to maintain objectivity. The exchange of opinions also substantially enriched the qualitative analysis and prevented one-sided interpretations of observed patterns of behavior. Of particular note are the work group discussions conducted with members of the Acholi ethnic group around the interpretation of observed mother and child behaviors. In contrast to recommendations related to member checking (Birt et al., 2016), we didn’t return our findings to participants of the study. The sole inclusion of local team members in workgroup discussions, however, simplified the implementation related to confidentiality, logistical issues (e.g., transportation to the office), and documentation (e.g., team members are literate and took their own notes, when suitable). In addition, it facilitated the implementation of regular meetings over an extended period of 7 weeks, which in turn allowed for more comprehensive and detailed discussions. Another benefit of our approach is related to the ethical issue of (group) coercion (Birt et al., 2016). Due to their long-standing work with vivo Uganda and their involvement in a variety of research projects with international teams, our local team members were used to the role of translating cultural concepts and teaching about cultural values. In addition, the first author emphasized her position of a student who wanted to learn about parenting and mother–child interactions in northern Uganda and actively encouraged disconfirming voices. Despite the named advantages, our local team members cannot be seen as representative for people living in northern Uganda due to their professional qualification and their high exposure to Western cultures. This fact most likely had an effect on their feedback regarding the coding manual.

The long process of learning and multi-cultural exchange set the stage for the development of the elaborated and comprehensive coding manual, which included very detailed culturally adapted code definitions, decision rules and behavioral examples drawn from the data. We were able to demonstrate the practical applicability of the coding manual during trainings. Members of our international workgroup were able to reliably employ the codes even if they were unfamiliar with the northern Ugandan context and culture. Because of findings suggesting the influence of ethnocentric perceptions on the accuracy of coder ratings (Yasui and Dishion, 2008), this result was especially promising regarding the future applicability of the coding scheme.

However, due to the high differentiation of the coding system as well as the logistical requirements for the implementation of the described behavioral observations, the feasibility of this method in post-war contexts may also be questioned. This matter might arise primarily because we did not include our local employees in the actual coding of the video tapes. In fact, due to a lack of resources, we had to decide against the training of our local partners in the application of the coding system. The very detailed code definitions, decision rules and behavior examples in the manual facilitate the learning of the different codes. However, a reliable differentiation of the individual codes can only be achieved if raters have enough time on hand to familiarize themselves with the manual and participate in appropriate training. In our opinion, this fact does not prevent the applicability of the presented method in a post-war context. We understand the developed manual as a guideline and endorse a flexible adaptation of the methodology to the existing conditions on site. If sufficient logistical and financial resources are available, all codes described can be employed. However, individuals and organizations, who intend to develop culture-sensitive and evidence-based (parenting) interventions, could also decide to only use the coding manual to familiarize themselves with typical mother–child interactions in northern Uganda. The manual can also serve as a prototype for future research on behavioral observations in post-conflict settings. Depending on the fields of application, the research questions, and available resources, individual codes can be selected from the manual or more global dimensions can be developed based on the described codes. In addition, the use of global affect codes can be recommended for a culture- and context-sensitive and at the same time resource-saving assessment of behavioral observations.

### Limitations

Notwithstanding its innovative nature, the present study was also subject to a number of limitations. One shortcoming refers to the selected sample. Participants were recruited from the narrowly defined area of Gulu town. Despite efforts to vary factors of interest potentially influencing parenting behaviors in northern Uganda such as maternal educational levels and war-exposure, the generalizability of findings across the Acholi ethnicity and the applicability to rural areas may be questionable. Besides, the sole reliance on biological mothers as primary guardians due to methodological considerations has its drawbacks. In collectivist cultures, apart from biological parents, community and family members play an important role in the socialization of children. In addition, due to the civil war in northern Uganda, many children are orphans and are raised by older siblings or members of the extended family (Ministry of Gender Labour and Social Development, 2009). The exclusion of primary guardians other than biological parents may have reduced confounding variables but may also have limited the informative value of the present study.

The conduct of behavioral observations in a laboratory setting is critically discussed. Authors argue that the artificiality of laboratory settings affects the authenticity of behaviors by participants and thus, the quality of the collected data (Gardner, 2000). Besides, despite extensive efforts to provide a culturally appropriate, natural and comfortable environment, the video setting and the invitation to the office of a humanitarian organization may have made the study appointment highly official. For instance, most mothers came to their appointments neatly dressed in their Sunday clothes. In addition, our local team members mentioned the suspicion that mothers put much effort in presenting themselves and their children from their best side. Grolnick et al. (2002) found that mothers in a high-pressure, ego-involving condition (children had to meet particular standards) exhibited more controlling parenting during a poem task with their children than did mothers in a low-pressure, non-ego involving condition. Even though not intended, a focus on good performance evoked by the environment may have influenced the patterns of behavior found in the present study. Also, affect codes show that both, mothers and children, were very frequently nervous. This finding might as well be related to the laboratory setting. One way to scrutinize these potentially interfering influences in future would be to directly ask participants’ perception of the authenticity of the setting and their own behaviors. In addition, the conduct of behavioral observations at participants’ homes would be highly valuable.

Even though the problem solving task is a widely and validly used structured interaction task, the mere reliance on one structured interaction task for the development of the coding system may have promoted the occurrence of behaviors and interactional patterns that were specific to characteristics of the task. Blacher et al. (2013), for instance, reported more negative parenting in a structured interaction task (problem solving task) compared to an unstructured interaction task (e.g., free play). Thus, future studies should verify findings by applying the coding system to the remaining two game activities and two child topics.

The predefined sequence of interaction tasks was the result of previous meetings with our local team members. They suspected that the problem solving task would influence all further interactions, as mothers would dominate the interaction with their children, continue to advise them and hardly let their children speak. In order to increase the probability that children would be able to mention their issues, we decided to conduct the Parent Issue after the discussion of the two child topics. However, the preceding structured interaction tasks might have impacted the quality and nature of the interaction during the problem solving discussion. For instance, negative emotions caused by the previous discussion may have increased the frequency of coercive parenting practices. Due to the rather demanding procedure, it is also conceivable that especially younger children were already tired at the time of the problem-solving task and therefore participated less actively in the conversation. However, it may also have been advantageous that the task selected was the forth in the sequence. Mother-child dyads already had time to get used to the setting. Therefore they might have behaved more authentically than at the beginning of the behavioral observations. In order to examine these contrary assumptions more closely, future studies could vary the sequence of interaction tasks, reduce the number of different interaction tasks or increase the duration of individual interaction tasks.

## Conclusion

Despite the limitations, this study represents an important extension of previous research on parenting and parent–child interactions in post-war environments. It forms the basis for the culture- and context-sensitive use of behavioral observations of mothers and children in northern Uganda. The developed coding system enables a very detailed, culturally sensitive analysis of positive and coercive interaction patterns and their relation to war and child development. The resulting observational data can significantly expand previous findings on the effects of war on family dynamics and provide important clues for the development and evaluation of family-oriented prevention and intervention programs.

## Data Availability Statement

The datasets generated for this study are available on request to the corresponding author.

## Ethics Statement

The ethics committee of the German Research Foundation (DFG), the ethics committee of Gulu University in Uganda and the National Council for Science and Technology (UNCST) in Uganda approved of the study protocol. Written informed consent to participate in this study was provided by the female guardians. In addition, children’s assent was obtained.

## Author Contributions

All authors made substantial contributions to conception and design of the study, contributed to manuscript revision, and read and approved the submitted version. JM and RS conducted data acquisition. JM and EW performed the qualitative data analysis and organized the database. JM performed further statistical analysis and wrote the manuscript.

## Conflict of Interest

The authors declare that the research was conducted in the absence of any commercial or financial relationships that could be construed as a potential conflict of interest.
